# A new diatom species *P*. *hallegraeffii* sp. nov. belonging to the toxic genus *Pseudo-nitzschia* (Bacillariophyceae) from the East Australian Current

**DOI:** 10.1371/journal.pone.0195622

**Published:** 2018-04-12

**Authors:** Penelope A. Ajani, Arjun Verma, Malwenn Lassudrie, Martina A. Doblin, Shauna A. Murray

**Affiliations:** Climate Change Cluster (C3), University of Technology Sydney, Broadway, NSW, Australia; Department of Bionanotechnology Center for Nanosciences and Nanotechnology Universidad Nacional Autónoma de México, MEXICO

## Abstract

A new species belonging to the toxin producing diatom genus *Pseudo-nitzschia*, *P*. *hallegraeffii* sp. nov., is delineated and described from the East Australian Current (EAC). Clonal cultures were established by single cell isolation from phytoplankton net hauls collected as part of a research expedition in the EAC region in 2016 on the *RV Investigator*. Cultures were assessed for their morphological and genetic characteristics, their sexual compatibility with other *Pseudo-nitzschia* species, and their ability to produce domoic acid. Light and transmission electron microscopy revealed cells which differed from their closest relatives by their cell width, rows of poroids, girdle band structure and density of band straie. Phylogenetic analyses based on sequencing of nuclear-encoded ribosomal deoxyribonucleic acid (rDNA) regions showed this novel genotype clustered within the *P*. *delicatissima* complex, but formed a discrete clade from its closest relatives *P*. *dolorosa*, *P*. *simulans*, *P*. *micropora* and *P*. *delicatissima*. Complementary base changes (CBCs) were observed in the secondary structure of the 3’ nuclear ribosomal transcribed spacer sequence region (ITS2) between *P*. *hallegraeffii* sp. nov. and its closest related taxa, *P*. *simulans* and *P*. *dolorosa*. Under laboratory conditions, and in the absence of any zooplankton cues, strains of *P*. *hallegraeffii* sp. nov. did not produce domoic acid (DA) and were not sexually compatible with any other *Pseudo-nitzschia* clones tested. A total of 18 *Pseudo-nitzschia* species, including three confirmed toxigenic species (*P*. *cuspidata*, *P*. *multistriata* and *P*. *australis*) have now been unequivocally confirmed from eastern Australia.

## Introduction

*Pseudo-nitzschia* Peragallo is a pennate diatom genus with global marine distribution [1]. Of the 49 species described to date, 24 have been found to produce domoic acid (DA) [2, 3], a potent neurotoxin which can accumulate in the marine food web and cause both ecosystem and human health effects [4]. It is hypothesised however, that under the right conditions (physical/chemical/biological interactions), all species of *Pseudo-nitzschia* may produce DA [5], and for this reason the routine monitoring of *Pseudo-nitzschia* cell densities and the concentration of the toxic compound DA is the focus of many seafood safety programs globally.

Identification of *Pseudo-nitzschia* to species level is complex, and is reliant on the investigation of intricate morphological traits, molecular markers and mating compatibility [6–13]. Key morphological features used to distinguish species include valve width, presence/absence of a central interspace, density of fibulae and striae, poroid number and arrangement, and cingular band structure [9, 14–16]. These taxonomically informative characteristics however, are not clearly demarcated between closely related or “cryptic” species, and therefore provide only one line of evidence for species resolution.

Molecular verification is also required for *Pseudo-nitzschia* species discrimination, with the most commonly used genetic markers being the internal transcribed spacer (ITS) and the D1-D3 regions of the large subunits (LSU) rDNA genes. Additionally, the secondary structure of the ITS2 region has become extensively used to predict reproductive incompatibility and genetic divergence between species [7, 9, 17]. Both the presence of compensatory base changes (CBCs) and hemi-CBCs (HCBCs) in the conserved regions of the ITS2 secondary structure helices [17] are used as a proxy for differentiation. The advent of these molecular markers two decades ago has, in fact, seen many new *Pseudo-nitzschia* species identified [6, 9–11, 17–21].

Using the combination of both genetic differences in ‘marker’ regions and morphological ultrastructure, eleven new species have been described in the past five years alone. From Malaysian Borneo [7, 13]—*P*. *circumpora* H. C. Lim, C. P. Leaw & P. T. Lim, *P*. *bipertita* S.T. Teng, H. C. Lim & C.P. Leaw and *P*. *limii* S.T. Teng, H. C. Lim & C.P. Leaw. From the Strait of Malacca Malaysia [8, 12]—*P*. *batesiana* H. C. Lim, S. T. Teng, C. P. Leaw & P. T. Lim, *P*. *lundolmiae* H. C. Lim, S. T. Teng, C. P. Leaw & P. T. Lim, *P*. *fukuyoi* H. C. Lim, S. T. Teng, C. P. Leaw & P. T. Lim, *P*. *kodamae* S.T. Teng, H. C. Lim, C.P. Leaw & P. T. Lim and *P*. *sabit* S.T. Teng, H. C. Lim, P. T. Lim & C.P. Leaw. Finally, from Bilbao estuary Spain [22]- *P*. *plurisecta* Orive & Perez-Aicua and *P*. *abrensis* Orive & Perez-Aicua and most recently *P*. *simulans* from Chinese waters [3]. Four of these species are confirmed producers of domoic acid: *P*. *kodamae* [12], *P*. *plurisecta* [22] and *P*. *fukuyoi* [23] and *P*. *simulans* [3].

Seventeen species belonging to the genus *Pseudo-nitzschia* have been identified thus far in Australia [24–29]. This genus has been identified as a dominant member of the phytoplankton community in both the coastal upwelling regions and estuarine systems of eastern Australia [26, 30–33]. Furthermore, three species have tested positive for domoic acid production in Australia (*P*. *australis*, *P*. *cuspidata* and *P*. *multistriata)* [26, 29].

The physical oceanography of Australia’s east coast is dominated by the western boundary current (WBC), the East Australian Current (EAC). The EAC redistributes low nutrient, warm tropical waters from the Coral Sea into temperate latitudes, and is generally weak compared with other WBCs [34]. A series of mesoscale eddies are associated with the EAC, and they interact with coastal upwelling provinces to produce a highly energetic, dynamic and complex coastal circulation [35]. In austral spring 2016, a scientific expedition was commenced on board the *RV Investigator* which offered a unique opportunity to sample microbial communities both within the EAC and its associated oceanic eddies. Arising from this expedition, we here delineate and describe a novel, potentially toxigenic diatom species *Pseudo-nitzschia hallegraeffii* sp. nov. isolated from the East Australian Current.

## Materials and methods

### Phytoplankton collection and water mass characteristics

**Fig 1:**
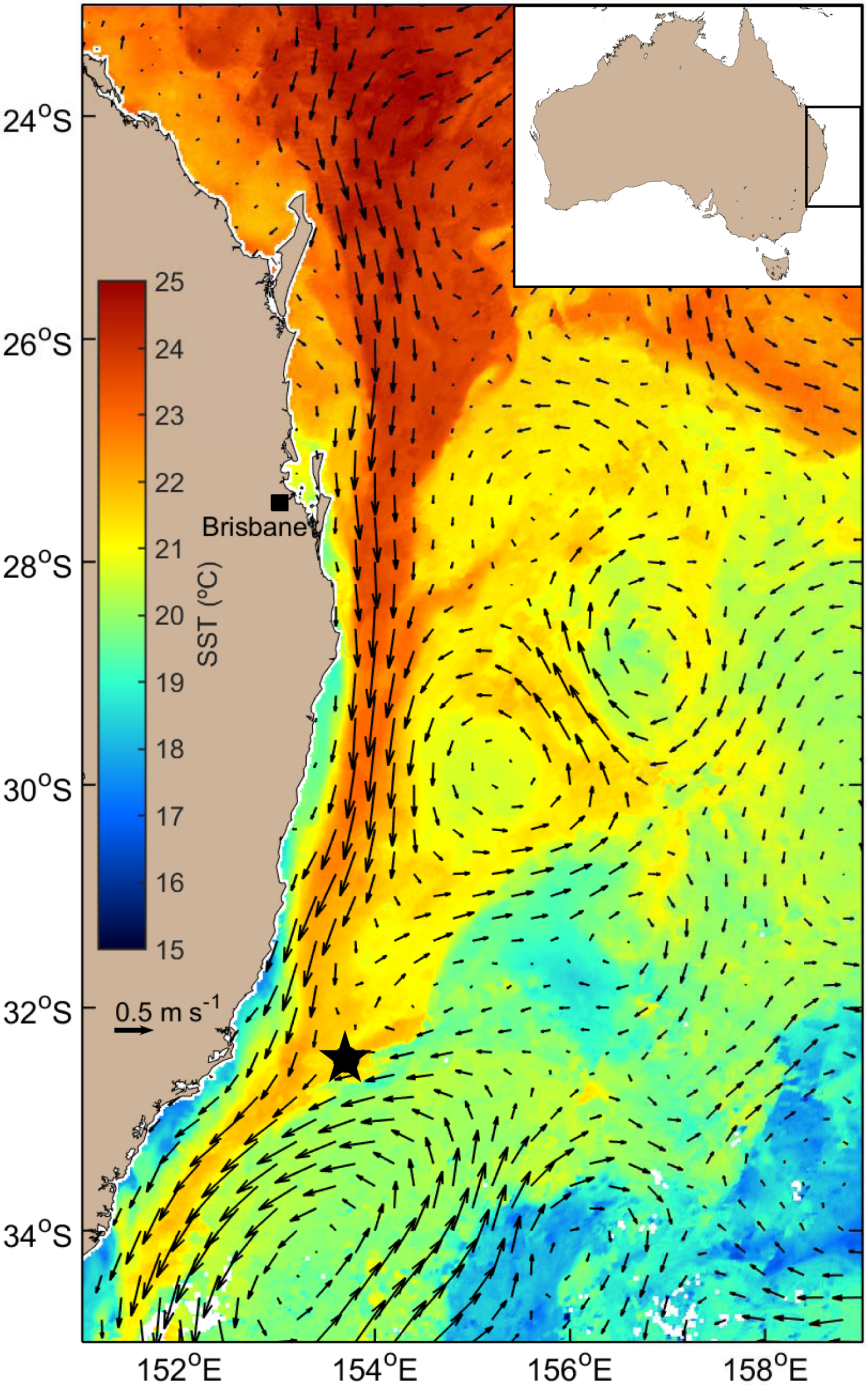
Map of the East Australian Current (EAC) as shown travelling south along the south-eastern Australian coastline and indicated by the warm sea-surface temperature. Station CTD44 (- 32.465°N, 153.705°S) is shown by black star. Sea surface data was compiled using the average highest available quality sea surface temperatures data from 12 to 17 September 2016 (NOAA-19 MOS—SRS Satellite—SST L3S - 06 day composite—day and night time composite) and eastward geostrophic current velocity data of 17 September 2016 (IMOS–Ocean Current—Gridded sea level anomaly—Near real time) (IMOS, 2016a and b).

Water samples were collected during the oceanographic voyage IN2016_V04 on board the Marine National Facility *RV Investigator* managed by the Commonwealth Scientific and Industrial Research Organisation (CSIRO). Samples yielding isolates were derived from one station located along the coast of New South Wales, Australia (Fig 1). At this site a phytoplankton sample was taken by hauling a 20 μm mesh net (245 mm diameter, 1.2 m length) with an attached 150 mL plastic jar to a depth of 20 m. From this net haul a 50 mL subsample was preserved using Lugols iodine and stored at 4°C before microscopic examination for phytoplankton community composition. Using a Sedgwick rafter cell the dominant taxa were enumerated from each net haul by counting up to 100 cells using a Nikon Eclipse TS100 inverted microscope (≤ 400x mag). The abundance of these taxa was considered semi-quantitative because of known biases associated with net sampling (Sournia, 1978). The remaining 100 mL of unfixed sample was filtered through a 100 μm mesh to remove meso-zooplankton and incubated on-board at 21°C under low illumination (~30 μmol photons m^-2^ s^-1^) before being brought back to the laboratory for single-cell isolation of the diatom *Pseudo-nitzschia*.

Environmental data were acquired by the CSIRO Oceans and Atmosphere Hydrochemistry Team. A vertical profile of temperature (SBE3T S/N #4208, Sea-Bird Scientific, USA), salinity (measured as conductivity SBE4C S/N #2808, Sea-Bird Scientific, USA), dissolved oxygen (SBE43 S/N #3154, Sea-Bird Scientific, USA) and chlorophyll-a fluorescence (Aquatrack III—06-5941-001, Chelsea Technologies Group, UK), was measured using a CTD (conductivity-temperature-depth)-profiler. Sensors were calibrated by on-board analyses using a Guildline Autosal Laboratory Salinometer 8400(B)–SN 71611, and an automated Photometric Oxygen system (Scripps Institute of Oceanography).

Dissolved nutrient analyses (phosphate, silicate, nitrite, nitrate and ammonium) were analysed from Niskin bottle samples. A segmented flow auto-analyser Seal AA3HR was used, following the standard operational procedures (SOP 001–004) modified from published methods by the CSIRO Oceans and Atmosphere Hydrochemistry Team to optimise nutrient analysis at sea. Briefly, phosphate was determined using the molybdenum blue method, based on Murphy and Riley [36] with modifications from the NIOZ-SGNOS Practical Workshop (2012). Silicate was also measured using the molybdenum blue method, and nitrite and nitrate using the Cu-Cd reduction–Naphthylenediamine photometric method, both based on Armstrong et al [37]. Ammonium was analysed using the ortho-phtaldiadehyde method based on Kérouel and Aminot [38]. The accuracy of nutrient analysis was determined by analysing a certified reference material produced by KANSO, Japan. The RMNS Lot CA (produced 22/02/2013) was measured four times in every analytical run. The RMNS Lot CD (produced 08/04/2015) was analysed twice alongside the CA Lot. RMNS results were converted from μmol kg^-1^ to μmol l^-1^ at 21°C.

### Pseudo-nitzschia isolation and culture maintenance

Non-axenic clonal cultures were established by isolation of *Pseudo-nitzschia* cells using drawn out glass pipettes (micropipettes) and a Nikon Eclipse TS100 inverted microscope (≤ 400x magnification) and transferred into 24 multi-well culture plates (Corning Inc. Durham, USA) containing 1 mL f/2 medium [39]. These well plates were kept at 16°C– 18°C under a photon flux of 60–100 μmol photon m^-2^ s^-1^ on a 12/12 hour dark/light cycle (white fluorescent tubes) and checked every alternate day. After 1 week, viable cultures were transferred to 70 mL gamma sterile polystyrene containers with polyethylene caps (Thermo Fisher Scientific, Australia, Pty.) and maintained in the same conditions. One milliliter of culture from each strain was transferred into fresh media every two weeks to establish healthy and exponentially growing monocultures over the duration of the study. On day 14 (late stationary phase) *Pseudo-nitzschia* cells were harvested for light (LM) and transmission electron microscopy (TEM) examination, DNA sequencing based on the large subunit (LSU) and internal transcribed spacer (ITS1-5.8S-ITS2) regions of the ribosomal DNA, and toxicity determination by liquid chromatography–mass spectrometry (LC-MS/MS) for the presence of DA.

### Morphological examination

*Pseudo-nitzschia* cultures were examined using a Nikon Eclipse TS100 inverted light microscope (LM) equipped with a Lumenera Infinity 3 digital camera (Ottawa, Canada) to a maximum magnification of ×400. For TEM examination, cultures were preserved in Lugol’s iodine prior to cleaning using the method of Hasle and Fryxell [40]. Once cleaned, 3 μl of each strain was placed on formvar-coated copper grids and loaded into a FEI Tecnai T20 TEM (LaB6), operated at a high tension of 120 kV and equipped with a Gatan 894 CCD 2k × 2k camera. Images for cell shape were obtained using LM, while all other frustule characteristics and morphometrics were obtained from TEM images, quantified using Image J1 (https://imagej.net/ImageJ1).

### DNA extraction and PCR amplification

DNA was extracted using a modified 3% CTAB buffer (100 mM Tris-HCl pH 8; 20mM EDTA pH 8; 1.4 M NaCl; 0.5% beta-mercaptoethanol) [41]. In summary, 30 mL of dense culture was centrifuged at 1000 *g* for 5 min at room temperature and the resulting pellet placed into 1 mL of CTAB buffer and incubated in a heat block at 68°C for one hour. The aqueous layer was then separated using chloroform and precipitated in isopropanol and sodium acetate. The DNA pellet was then washed with ethanol and vacuum dried to remove any traces of ethanol. Sterile Milli-Q water was added to the DNA pellet and the sample were stored at -20°C prior to PCR reactions. The extracted DNA was visualised on agarose gel and quantified using a Nanodrop ND-1000 (NanoDrop Technologies, Wilmington, USA) [41].

The partial D1-D3 regions of the LSU rRNA gene, the internal transcribed spacer regions and 5.8S rRNA gene (ITS1-5.8S-ITS 2) were amplified and sequenced as described in Verma et al. [41]. All PCR reactions contained 12.5 μL 2x Immomix (Bioline, Sydney, Australia), 7.5 pmol of each primer (Table 1), 1 μg μL^−1^ of BSA (Biolabs, Arundel, Australia), 1 μL of template DNA and PCR grade water to give the final volume of 25 μL. Thermocycling conditions consisted of an initial denaturing step of 95°C for 10 min, followed by 30 cycles of 95°C for 20 s, 58°C for 30 s, 72°C for 1.5 min and a final extension of 72°C for 7 min. PCR products were purified with DNA Clean and Concentrator (ZymoResearch, Irvine, USA) according to the manufacturer’s protocol. The PCR products were sequenced using a commercial service (Macrogen Inc., Seoul, Korea).

**Table 1:** Primers used for the amplification of the LSU and ITS/5.8 regions of rDNA from clonal cultures of *Pseudo-nitzschia* established in this study.

Primer	Primer sequence	Target region	Direction	Reference
DIR	5'-ACC CGC TGA ATT TAA GCA TA-3'	28S (D1-D3)	Forward	[42]
D3B	5'-TCG GAG GGA ACC AGC TAC TA-3'	28S (D1-D3)	Reverse	[43]
ITSA	5'-GTA ACA AGG THT CCG TAG GT-3'	ITS1-5.8S-ITS2	Forward	[44]
ITSR	5'-ATA TGC TTA AAT TCA GCG GGT-3'	ITS1-5.8S-ITS2	Reverse	[44, 45]
ITSF	5'-TTC CGT AGG TGA ACC TGC GG -3'	ITS1-5.8S-ITS2	Forward	[45]
PnITSF	5'-ACT TTC AGC GGT GGA TGT CTA -3'	5.8-ITS2	Forward	[46]
PnITSR	5'-CTT GAT CTG AGA TCC GGA ATT-3'	5.8-ITS2	Reverse	[46]

### Sequence analysis and phylogenetic reconstruction

Analyses on the D1-D3 region of LSU rDNA and ITS-5.8S were conducted separately. The forward and reverse sequences were trimmed, aligned and visually refined using BioEdit v7.2.5 [47]. The obtained sequences were aligned with reference sequences retrieved from GenBank (S1 Table). Multiple sequence alignments were performed using ClustalW v1.6 program as implemented in MEGA v7 and manual inspection [48]. All positions containing gaps and missing data were eliminated. Phylogenetic analyses were performed using both maximum likelihood (ML) and Bayesian inference (BI) approaches. ML trees were inferred in MEGA v7 using general time reversible (GTR) + gamma (G) + inversions (I) substitution model for ITS-5.8S sequence analyses. Substitution models were selected for each dataset based on lowest Bayesian Information criterion (BiC) as a measure of the relative quality of the models. Nodal support of the ML tree was estimated via bootstrap algorithm with 1000 replications. Bayesian analysis was performed using MrBayes v3.2.2 [49] as implemented in Geneious v7 [50] using GTR + G model for all analyses. Four independent Markov Chain Monte Carlo simulations were run simultaneously for 2 x10^6^ generations. Trees were sampled every 1000 generations and 1000 trees were discarded as burn-in. Genetic distance (pairwise uncorrected *p*-distance) was estimated from the ITS/5.8S and D1-D3 LSU rDNA sequences using the *p*-distance model and bootstrap procedure (1000 replicates) in MEGA v7 [48].

### Modelling ITS2 secondary structure

The ITS2 region was identified and delimited based on alignment with *Pseudo-nitzschia dolorosa* strains BP3 and 300 (GenBank accession numbers DQ336151 and DQ336153 respectively). After removing the 3’ and 5’ ribosomal termini, the annotated ITS2 sequences were aligned using ClustalW in MEGA v7 and adjusted manually. The secondary structures of the ITS2 region from *P*. *hallegraeffii* strains were predicted using Mfold using the default parameters [51]. RNA transcript folding of ITS2 for the two *Pseudo-nitzschia* strains in this study was predicted by the ITS2 Database V using default settings (http://its2.bioapps.biozentrum.uni-wuerzburg.de/) with ITS2 PAM50 matrix chosen and the percentage of transfer helices at 75% similarity selected [52]. Structures were visualized using VARNA and compensatory base changes (CBCs) and hemi-CBCs were identified and compared to *P*. *simulans* MC984 (GenBank accession number: MF374772) and *P*. *dolorosa* 300 (GenBank accession number: DQ336153) using 4SALE [53–55].

### Mating experiments

Mating experiments to test for reproductive isolation of *P*. *hallegraeffii* sp. nov. were carried out during the exponential growth phase between all possible pair-wise combinations of available *Pseudo-nitzschia* strains: *P*. *hallegraeffii* CTD44_2 and *P*. *hallegraeffii* CTD44_3 (isolated during the present study); *P*. *simulans* (Strain CTD#49-200916-1); and *P*. *pungens* var. *averensis* (Strain DER300816-1). One mL of each pair-wise culture was combined a 12-multiwell culture plate (Corning Inc. Durham, USA) at a starting concentration of ~5,000 cells L^-1^ per strain. Each pair-wise combination was prepared in triplicate and maintained as per the culture conditions outlined above. Mixed cultures were examined daily using light microscopy for the presence of sexual stages (gametes, zygotes and/or auxospores) until they reached stationary phase.

### Toxin determination

A Thermo Scientific™ Q EXACTIVE™ high resolution mass-spectrometer equipped with an electrospray ionization source was used for the detection of DA. A certified standard solution of domoic acid (DA) was purchased from National Research Council of Canada (NRC, Halifax, Nova Scotia, Canada). Six calibration standards were prepared by diluting the standard solution so that the concentration ranges from 1 to 200 ng m^-l^.

*Pseudo-nitzschia* cultures were harvested in late stationary phase by centrifugation (50 mL; 1500 g; 5 min) and cell pellets freeze dried and stored at 4°C before toxin extraction [56] and DA analysis. The pellet was dried down under nitrogen (flow) and re-suspended in 50 μL of 90% MeOH. The solution was vortexed for 1 min followed by sonication for 1 min. The solution was then centrifuged for 5 min at 2283 g and the supernatant was used for chromatographic separation on a Thermo Scientific™ ACCELA™ UPLC system using routine analysis method used at the Sydney Institute of Marine Science (SIMS) (unpublished). Analysis was performed using an Acquity UPLC BEH Shield RP18 1.7 μm 2.1 x 50 mm column with an injection volume of 5 μL. The mobile phases used were A (water), B (acetonitrile/water/formic acid at 475: 25: 1.5: 0.12 v/v/v). The initial condition started with mobile phase A at a flow rate of 450 μl min^-1^ and was held for 0.5 min. The condition was then linearly changed over 0.5 min from A:B (100:0) to A:B (85:15), then from A (85:15) to A:B (65:35) over 0.6 min and then from A: B (65:35) to A:B (25:75) over 3.4 min. The gradient was then changed to 100% B while the flow rate was gradually changed from 450 μl min^-1^ to 800 μl min^-1^ over 2.0 min. The condition was then set to initial condition 100% A and flow rate of 450 μl min^-1^ in 1 min, and then the column was re-equilibrated for 2 min before running the next sample. DA was reported as ng mL^-1^ with the limit of detection for the analyses reported as 0.1 ng mL^-1^.

## Results

### Phytoplankton cultures and water mass characteristics

Two clonal *Pseudo-nitzschia* cultures were successfully isolated from the *RV Investigator* expedition (IN2016_V04) from strains CTD44_2 and CTD44_3 (-32.465°N, 153.705°S, CTD44, Table 2). This station was ~110 km offshore (bottom depth >4700 m), northeast of Newcastle, NSW, in the EAC. The characteristics of the water mass sampled are summarised in Table 3. The phytoplankton community at this station was dominated by the diatom taxa *Chaetoceros* spp., *Pseudo-nitzschia* spp., *Cylindrotheca (Ceratoneis) closterium*, *Climacodium frauenfeldianum* and *Thalassiosira* spp. (in order of numerical dominance).

**Table 2:** List of *Pseudo-nitzschia* strains, clone designation, collection location and date/time sampled, as well as accession numbers for the LSU rDNA and ITS/5.8 rDNA sequences established in the present study.

Species	Clone Designation	Location Sampled	Date/Time Sampled	Genbank Strain ID	LSU Accession No.	ITS Accession No.
*P*. *hallegraeffii* sp. nov.	CTD44_2	East Coast of Australia (-32.465°N, 153.705°S)	17/09/2016 04:35 UTC	CTD44_2	MF044024	MF044023
	CTD44_3	East Coast of Australia (-32.465°N, 53.705°S)	17/09/2016 04:35 UTC	CTD44_3	MF044022	MF044025

**Table 3:** Location, date, time and physico-chemical characteristics of the seawater sampled at the surface (3-5m depth) and at the deepest sampling point (20–20.5m) on board the *RV Investigator*.

Station	Latitude	Longitude	Date (UTC)	Time (UTC)	Day / night	Depth (m)	Temperature (°C)	Salinity	Dissolved Oxygen (μmol L^-1^)	Chlorophyll-a Fluorescence (RFU)	Phosphate (μmol L^-1^)	Silicate (μmol L^-1^)	Nitrate (μmol L^-1^)	Nitrite (μmol L^-1^)	Ammonia (μmol L^-1^)
CTD44	-32.465	153.705	17/09/2016	4:34	Day	3–5	21.33	35.78	223.1	24.45	0.13	0.4	0.299	0.091	0.05
						20–20.5	21.07	35.78	224.4	29.75	0.13	0.3	0.247	0.093	0.03

### Species description

**Fig 2:**
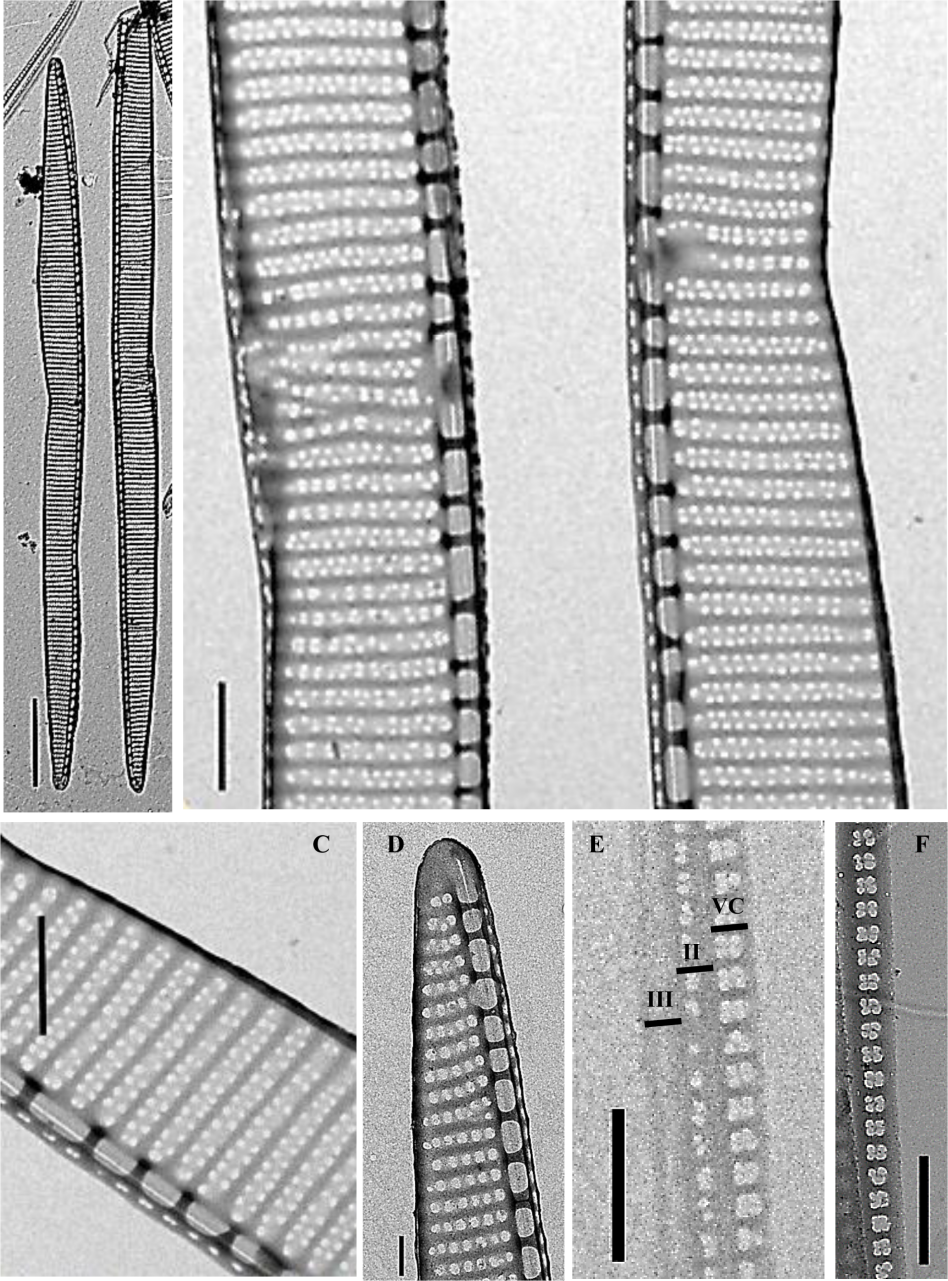
*Pseudo-nitzschia hallegraeffii* sp. nov. A-F) transmission electron microscopy; A) whole valves (scale bar 10 μm, strain CTD44_3); B. mid-valves showing large central interspace (scale bar 1 μm, strain CTD44_3); C) mid-valve showing interstriae, fibulae and two rows of poroids (scale bar 1 μm, strain CTD44_3); D) valve end (scale bar 0.5 μm, strain CTD44_2); E) cingulum girdle bands: V = valvocopula, II = second copula, III = third copula (scale bar 1 μm, strain CTD44_3); F) valvocopula showing poroid structure of two poroids wide and one or two poroids high (scale bar 1 μm, strain CTD44_3).

***Pseudo-nitzschia hallegraeffii*** Ajani, Verma et Murray (Fig 2; Table 4)

**Table 4:** Species and morphological information pertaining to *Pseudo-nitzschia delicatissima* complex including two strains of *Pseudo-nitzschia hallegraeffii* sp. nov. established in this study. Note: n = number of specimens observed; data are given as minimum and maximum range (above) and mean ± SD (below); nd = no data; # = no. poroids wide x no. poroids high–valvocopula pattern; followed by band II pattern.

Species/Strain ID	Valve Shape	Central interspace	Apical Axis (μm)	Transapical Axis (μm)	Interstriae per 10 μm	Fibulae per10μm	Rows of Poroids	Poroids per 1μm	Band Striae per 10μm	Band Striae Structure#	Reference
Strain CTD44_2	lanceolate, asymmetrical	+	25.6–44.3 (39±3.8) (n = 45)	2.2–3.0 (2.6±0.2) (n = 44)	36–40 (37.6±1.1)(n = 11)	16–22 (19.4±1.8)(n = 10)	2(1)	6–8 (6.7±0.6) (n = 15)	43–56 (46.8±6.2)(n = 4)	1–2 x 1–2 (n = 6), 2 x 1 (n = 6)	This study
Strain CTD44_3	lanceolate, asymmetrical	+	29.1–55.4 (43.5±6.7)(n = 45)	1.9–3.1 (2.4±0.3) (n = 43)	34–39 (36.2±1.4)(n = 15)	19–22 (20.0±1.0)(n = 15)	2(1)	6–8 (7.6±0.6) (n = 15)	46–50 (48±1.6) (n = 10)	1–2 x 1–2 (n = 9), 2 x 1 (n = 10)	This study
*P*. *simulans*	lanceolate/ sigmoidal in girdle view	+	37–49 (43.2±5.4)(n = 30)	1.8–2.1 (1.9±0.1) (n = 25)	34–44 37±3 (n = 30)	19–23 21±2 (n = 30)	1	5–7 (6±1) (n = 30)	40–55 (47±4) n = 12	2 x 2	[3]
*P*. *dolorosa* (5)	lanceolate, asymmetrical	+	30–59	2.5–3.0 (2.6±0.2)	30–36 (34.5±1.4)	18–22 (20.0±1.0)	1–2	5–8 (6.6±0.8)	40–44 (42.0±1.4)	2 x 3, 2 x 1	[11]
		+	42.4–43.0 (42.7±0.3)(n = 30)	1.8–2.1 (2.0±0.2) (n = 30)	35–37 (36.2±0.8)(n = 5)	21–22 (21.6±0.5)(n = 5)	1	5–6 (5.8±0.5) (n = 8)	nd	nd	[7]
*P*. *delicatissima* (20)	lanceolate	+	19–76	1.5–2.0 (1.8±0.2)	35–40 (36.8±1.5)	19–26 (21.4±1.6)	2	8–12 (10.1±1.2)	43–48 (44.2±1.6)	1 divided poroid	[11]
		+	nd	1.7–2.0 (1.8±0.1)	33–37 (34.7±1.2)	19–21 (19.8±1.0)		8–12 (10.4±1.1)	nd	nd	[22]
*P*. *decipiens* (5)	lanceolate	+	29–64	1.4–2.4 (1.9±0.3)	41–46 (43.2±1.2)	20–26 (24.0±1.4)	2	9–13 (11.4±1.2)	48–55 (51.8±1.7)	1 divided poroid	[11]
		+	41.8–49.1 (n = 10)	1.7–2.0 (n = 11)	43–47 (n = 13)	22–26 (n = 13)	2	8–13 (n = 24)	48–54 (n = 8)	2 x (1)2 (n = 23), 2 x 1–2 (n = 20)	[57]
*P*. *galaxaie*	lanceolate	+	19–50 (33.8±9.0)(n = 21)	1.1–1.6 (1.4±0.2) (n = 23)	55–70 (63.8±4.0) (n = 25)	17–28 (24.0±2.9)(n = 25)	nd	nd	63–68 (65.8±1.8)(n = 8)	nd	[58]
*P*. *micropora* (18)	lanceolate	-	33.1–36 (34.9±0.9)	1.8–2.3 (2.0±0.1)	42–50 (45.9±2.9)	23–30 (27.1±2.7)	2	9–13 (11±1)	54–60 (59.3±2.1)	2 x 2	[25]
			32.1–37 (34.6±2.5)(n = 30)	1.8–2.2 (2.0±0.2) (n = 31)	42–45 (43.4±1.7)(n = 5)	24–27 (25.6±1.8)(n = 5)	2	11–12 (11.3±0.8)(n = 6)	50–55	2 x 2	[7]

#### Diagnosis

Cells are asymmetrical and lanceolate in valve and girdle view, 25.6 to 55.4 μm long and 1.9 to 3.1 wide. Cells have a large central interspace towards the midpoint of the valve. The number of interstriae and fibulae in 10 μm are 34 to 40 and 16 to 22. Each stria is biseriate, but even within the same stria, poroids can sometimes merge to become uniseriate. When biseriate, poroids often form two opposite rows. Valve poroids are 6 to 8 per μm. The valvocopula contains 43 to 56 striae in 10 μm and is, in the pervalvar direction, 1–2 poroids wide and 1–2 poroids high. The second cingular band is 2 poroids wide and 1 poroid high while the third is unperforated.

#### Type locality

East Australian Current (-32.465°N, 153.705°S), east coast of Australia.

#### Holotype

Permanent slides of both strains (CTD44_2 UMACC No. 415; CTD44_3 UMACC No. 416: 5 replicates of each) have been deposited in the UMACC Algal Culture Collection, University of Malaya, Malaysia.

#### Etymology

This species is named in honour of Professor Gustaaf M. Hallegraeff for his outstanding contributions to the field of harmful algal research, especially his ground-breaking work in documenting Australian phytoplankton and harmful algal species.

#### Molecular characterisation

Nucleotide sequences of the ITS/5.8 and LSU rDNA regions for both strains have been deposited in Genbank (NCBI) with accession numbers given in Table 2.

#### Description

Cells occurred as single cells or in stepped pairs with a cell overlap of ~ 1/9. Cells were asymmetrical and lanceolate with all cells tapering towards the tip in valve view (Fig 2A). A large central interspace was observed towards the middle of each cell (Fig 2B, Table 4). Cells had an apical axis ranging between 25.6 to 55.4 μm and a transapical axis range of 1.9 to 3.1 μm and contained 16–22 fibulae per 10 μm and 34–40 interstriae per 10 μm (Fig 2B and 2C, Table 4). Two rows (infrequently one row) of small poroid occlusions were observed, often with these varying rows of poroids within the same cell (Fig 2B and 2C, Table 4). In the central valve area, poroids numbered between 6 and 8 per μm. The tip of the valve appeared rounded in both girdle and valve views (Fig 2D valve view only). The cingulum on both the epitheca and hypotheca comprised three girdle bands, with the valvocopula observed to have 43–56 striae per 10 μm (Fig 2E, Table 4). The valvocopula also displayed a poroid arrangement of two rows wide and one to two rows high per stria (Fig 2E and 2F, Table 4), the second had a 2 x 1 poroid arrangement (Fig 2F, Table 4), and the third cingular band was unperforated (Fig 2F, Table 4).

### Toxicity

No domoic acid was detected in either strain analysed (CTD44_2 or CTD44_3) at a detection limit of 0.1 ng mL^-1^.

### Sequence analysis and phylogenetic reconstruction

**Fig 3:**
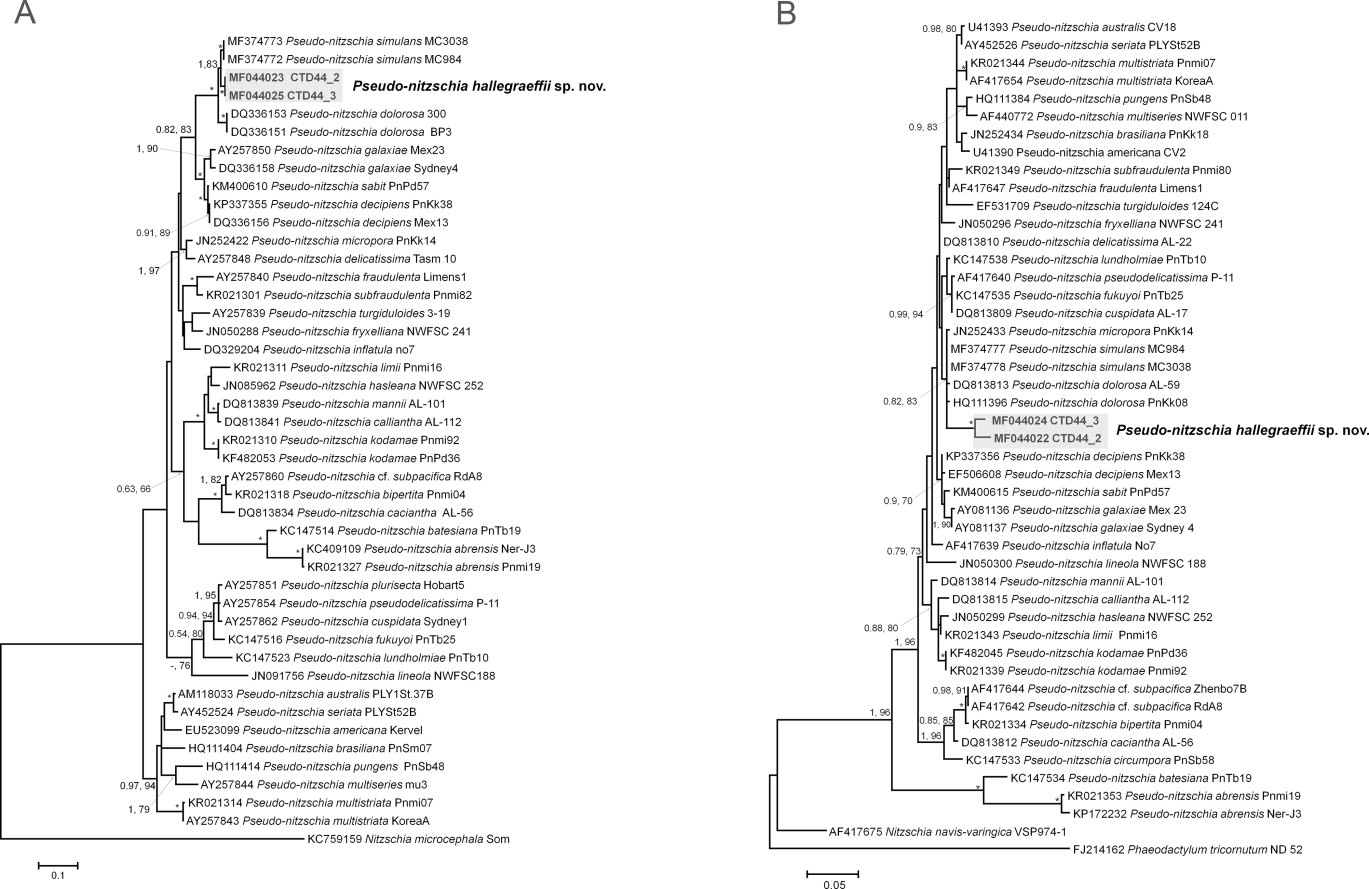
**A) *Pseudo-nitzschia* phylogenetic analyses based on the internal transcribed spacer (ITS1-5.8S-ITS2) regions of the nuclear encoded rDNA.** The tree is rooted using the outgroup *Nitzschia microcephala*; B). *Pseudo-nitzschia* phylogenetic analyses based on the large subunit (LSU) region of the nuclear encoded rDNA and rooted with the diatom outgroups *Nitzschia navis-varingica* and *Phaeodactylum tricornutum*. Shaded areas show the *P*. *hallegraeffii* strains that form novel and fully supported monophyletic clade. Numbers at nodes represent posterior probabilities from Bayesian Inference (BI) and bootstrap support values from Maximum Likelihood (ML) analyses based on 1000 pseudo-replicates. Bootstrap values only greater than 66% are represented in the figure. * represents 1, 100 support values for BI and ML respectively.

Phylogenetic trees were inferred using 47 sequences (805 base pairs) of the LSU rDNA and 45 sequences (1145 base pairs) of the ITS/5.8S rDNA (which included the two strains isolated in this study as well as outgroups) (S1 Table). Based on phylogenetic analyses using ML and BI methods, the topology recovered was similar to previously published phylogenies of the genus *Pseudo-nitzschia*. ML and BI analyses revealed the East Australian Current strains formed a novel and fully supported monophyletic clade (BI, ML = 1, 100) (Fig 3A and 3B). This new clade falls within the ‘*P*. *delicatissima* complex’ and is most closely related to *P*. *simulans* and followed by *P*. *dolorosa*. Higher divergence was found for the LSU D1-D3 rDNA sequences (0.022 ± 0.009) using pairwise uncorrected *p*-distances (1000 pseudo-replicates), compared to the ITS/5.8S sequences within *P*. *hallegraeffii* strains, whilst the genetic distance between *P*. *hallegraeffii* and *P*. *simulans* strains was 0.032–0.037 and 0.018 for LSU D1-D3 and ITS-5.8S rDNA regions respectively. Genetic distances between *P*. *dolorosa* and *P*. *hallegraeffii* were 0.034–0.039 and 0.036 for LSU and ITS-5.8S respectively (S2 and S3 Tables).

### Modelling ITS2 secondary structure

**Fig 4:**
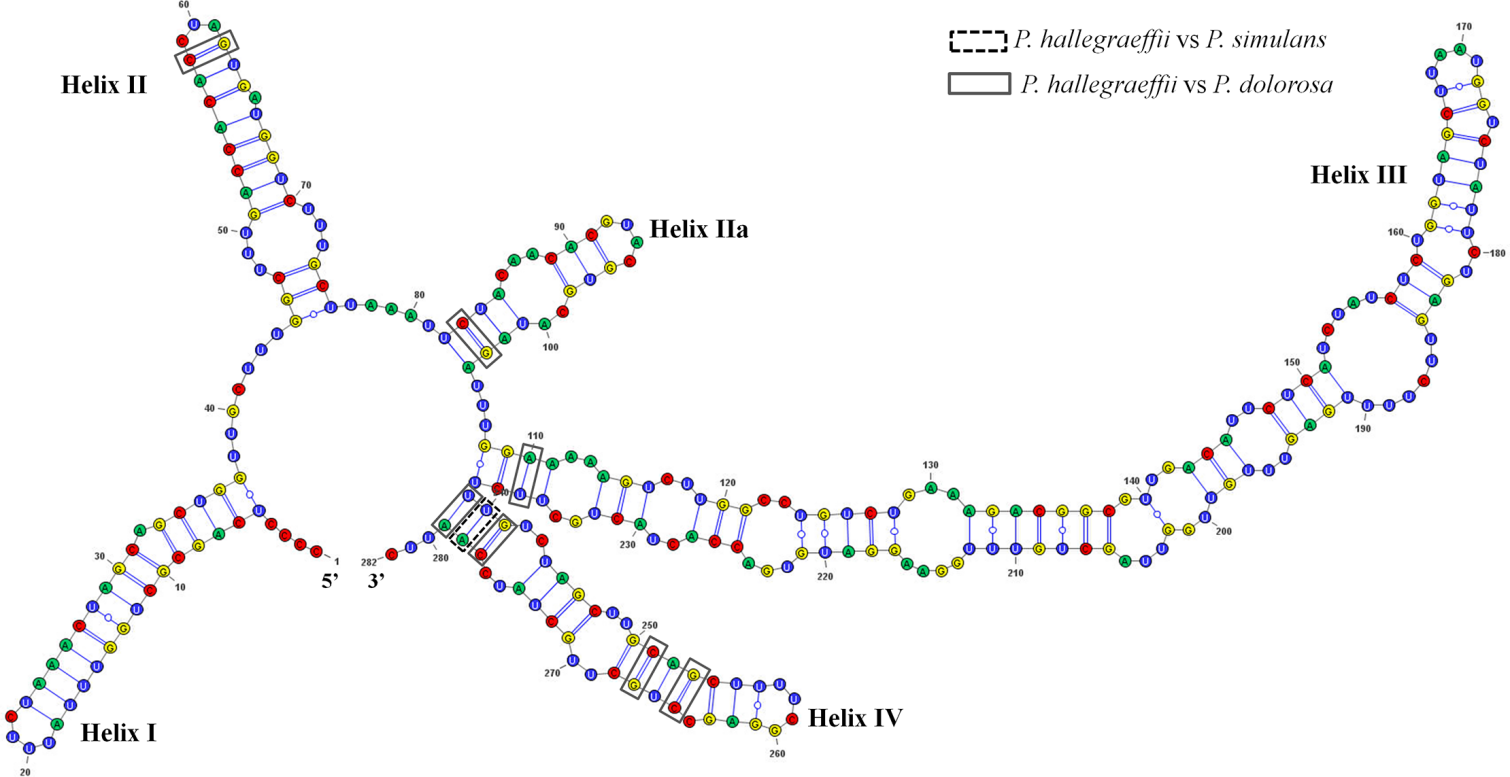
Predicted ITS2 structures of *P*. *hallegraeffi* sp. nov. (strain CTD44_2). Black dashed box represents CBCs between *P*. *hallegraeffii* vs *P*. *simulans*. Grey boxes represent CBCs between *P*. *hallegraeffii* vs. *P*. *dolorosa*. S1 Fig shows predicted ITS2 structure of A. *P*. *simulans* and B. *P*. *dolorosa*.

In modelling the ITS2 secondary structure it was found that four main helices (Helix I-IV) and one pseudo-helix (IIa) were recovered, as has been determined previously (Amato et al., 2007; Lim et al., 2012, 2013; Orive, 2013; Teng et al., 2015, 2016). Strains CTD44_2 and CTD44_3 revealed identical ITS2 structures (Fig 4). A large number of base pair substitutions in the ITS2 secondary structure transcript were observed between *P*. *hallegraeffii* sp. nov. and its closest relatives *P*. *simulans* and *P*. *dolorosa* (Fig 4). One complementary base changes (CBCs) and three hemi-CBCs were observed in the ITS2 structures between *P*. *hallegraeffii* and *P*. *simulans* whilst seven CBCs and six hemi-CBCs were observed in the ITS2 structures between *P*. *hallegraeffii* and *P*. *dolorosa* (Table 5).

**Table 5:** List of compensatory base changes (CBCs) and hemi-CBCs between *Pseudo-nitzschia hallegraeffii* sp. nov. and closely related taxa *P*. *dolorosa* and *P*. *simulans*.

Species	CBCs	Hemi CBCs
*Pseudo-nitzschia hallegraeffii* sp. nov. versu*s Pseudo-nitzschia dolorosa*	Helix II C-G↔U-A Helix IIa C-G↔U-A Helix III A-U↔G-C Helix IV U-A↔G-C Helix IV G-C↔U-A Helix IV C-G↔A-U Helix IV G-C↔A-U	Helix III U-A↔U-U Helix III U-A↔U-G Helix III G-C↔G-U Helix III U-A↔U-U Helix III G-U↔G-A Helix III U-A↔U-U
*Pseudo-nitzschia hallegraeffii* sp. nov. versus *Pseudo-nitzschia simulans*	Helix IV U-A↔G-C	Helix I U-A↔U-G Helix III G-U↔A-U Helix IV U-G↔C-G

### Mating experiments

No sexual stages (gametes, zygotes and/or auxospores) were noted in any of the mixed pair-wise cultures which were observed daily until day 8 when all innoculated cultures reached stationary phase (Table 6).

**Table 6:** Results of mating experiments between all pair-wise combinations of *Pseudo-nitzschia* strains available.

Strain name	*P*. *hallegraeffii* CTD44_2	*P*. *hallegraeffii* CTD44_3	*P*. *simulans* CTD#49-200916-1	*P*. *pungens* var. *aveirensis* DER300816-1
*P*. *hallegraeffii* CTD44_2	O (3)			
*P*. *hallegraeffii* CTD44_3	O (3)	O (3)	O (3)	O (3)
*P*. *simulans* CTD#49-200916-1	O (3)	O (3)	O (3)	O (3)
*P*. *pungens* var. *aveirensis* DER300816-1	O (3)	O (3)	O (3)	O (3)

O sexual reproduction was not observed in mixed culture; (3) number of replicates of each pair-wise combination.

## Discussion

In this study, multiple lines of evidence, including distinct morphological and genetic differences, as well as mating incompatibility with other species of *Pseudo-nitzschia*, provide convincing evidence that the two clonal isolates from an oceanographic research voyage from the East Australian Current represent a novel species, here designated *P*. *hallegraeffii*. *P*. *hallegraeffii* is found to be part of the ‘*Pseudo-nitzschia delicatissima* complex” which characteristically have cells < 3 um wide [59]. Other species identified from Australian coastal waters from this complex include *P*. *arenysensis*, *P*. *caciantha*, *P*. *calliantha*, *P*. *cuspidata*, *P*. *dolorosa*, *P*. *galaxiae*, *P*. *lineola*, *P*. *multistriata*, *P*. *micropora* and *P*. *hasleana* [24–29]. All other *Pseudo-nitzschia* species identified thus far from Australian waters are crudely placed in the ‘*Pseudo-nitzschia seriata* complex’ (mean valve width >3 μm) [5, 59]. These include *P*. *americana*, *P*. *australis*, *P*. *fraudulenta*, *P*. *heimii*, *P*. *multiseries*, *P*. *pungens*, and *P*. *subpacifica*.

Within the ‘*P*. *delicatissima* complex’, the valve shape and dimensions of *P*. *hallegraeffii*, the presence of a central interspace, the interstriae and fibulae density, and the varying rows of poroids, show similarity to *P*. *dolorosa* Lundholm et Moestrup. However, the valvocopula band structure (1–2 poroids wide and 1–2 poroids high) and higher density of band striae in *P*. *hallegraeffii* compared to *P*. *dolorosa*, clearly distinguish these two species. Similarly, *P*. *hallegraeffii* and *P*. *simulans* both share the presence of a central interspace and similar interstriae, fibulae and poroid density, yet *P*. *simulans* has only one row of poroids, a smaller transapical axis, and a differing valvocopula band structure compared to *P*. *hallegraeffii* (Table 4). *P*. *hallegraeffii*, *P*. *dolorosa and P*. *simulans* can in turn be clearly distinguished from other closely related species of the ‘*P*. *delicatissima* complex’ (*P*. *delicatissima*, *P*. *decipiens*, *P*. *galaxiae* and *P*. *micropora*), by having wider, asymmetric valves (*P*. *simulans* is sigmoidal in girdle view) and fewer poroids per μm compared to these other taxa. It is also noteworthy here, that in the absence of molecular confirmation, Moschandreou et al. [60] isolated a strain from the north eastern Mediterranean that closely resembled *P*. *dolorosa* (strain (07)7A9 *P*. cf. *dolorosa*), yet its valvocopula band pattern varied from *P*. *dolorosa* (striae of 3 or 4 poroids in the pervalvar direction instead of 2–3 as in *P*. *dolorosa*, 2 or 3 poroids in the second band instead of 1 or 2 as in *P*. *dolorosa*, and its 1 or 2 poroids in the third band compared to none in *P*. *dolorosa*), indicating that there may be additional morphotypes to be discovered within the ‘*P*. *delicatissima* complex’.

We have also demonstrated that the delineation between *P*. *dolorosa*, *P*. *simulans* and *P*. *hallegraeffii* is well supported by phylogenetic analyses (ITS-5.8S and LSU) and genetic distance measures, thus reinforcing the partitioning of this species(s). In particular, the ITS secondary structure information clearly supports the hypothesis of a novel species, with one CBC (Helix IV) and three HCBCs (Helices I, III and IV) between *P*. *hallegraeffii* and *P*. *simulans* and seven CBCs (Helices II, IIa, III and IV) and six HCBCs (Helix III) between *P*. *hallegraeffii* sp. nov. and *P*. *dolorosa* respectively. The presence of CBCs between taxa has been extensively used to examine the differentiation between species, particularly in cases of cryptic and pseudo-cryptic species [61]. It has been demonstrated (amongst sexually and asexually reproducing plants and fungi taxa) that even in presence of one CBC (in the conserved helices of the ITS2 secondary structure, varying on taxa), there is a 93% reliability that the taxa constitute distinct species [62]. Within the genus *Pseudo-nitzschia*, assuming that all species within this group have evolved at approximately the same rate, the presence of HCBCs or even one CBC in the ITS2 (in combination with other morphological and mating differences), is sufficient to lineated between species. Moreover, the presence of HCBCs alone has been found to be consistent with sexual incompatibility and species delineation within the genus *Pseudo-nitzschia* [6, 8, 9, 13, 57, 61, 62].

Another proxy used for species diversification is genetic divergence, and whilst the divergence between *P*. *hallegraeffii* and *P*. *simulans* for the ITS-5.8S rDNA region was relatively low, there are many other examples of clearly delineated species of *Pseudo-nitzschia* with genetic distances being equivalent or lower than that found here. For example, the genetic distance between *P*. *cuspidata* and *P*. *pseudodelicatissima* is 0.01, between *P*. *plurisecta* (strain Hob 5) and *P*. *pseudodelicatissima* is 0.01, and between *P*. *plurisecta* and *P*. *cuspidata* is 0.015 (S2 Table). Moreover, the genetic distance between *P*. *hallegraeffii* and closely related species for the LSU D1-D3 region is significantly higher (0.037) than for many other *Pseudo-nitzschia* species delineations which are <0.03 (S3 Table).

Our mating experiments support the hypothesis that *P*. *hallegraeffii* is reproductively isolated, with no evidence of sexual reproduction observed even with its closest relative, *P*. *simulans*. In our experiments, no sexual reproduction was observed between the two strains of *P*. *hallegraeffii* which we isolated in this study. This may be due to several factors: 1) our experimental conditions were not sufficient for the onset of sexual reproduction; 2) the two strains are themselves reproductively isolated, and may represent unique populations, or 3), our strains of *P*. *hallegraeffii* were of the same mating types (*Pseudo-nitzschia* is heterothallic and requires different mating types for successful sexual reproduction [63]). The strain combinations for our mating experiments were established in the same way as has been previously demonstrated to modulate sexual reproduction in *Pseudo-nitzschia*, for example, cultures were in the exponential growth phase [64]; a starting cell concentration of 5000 cells mL^-1^ was used [64, 65]; and cultures were maintained in similar media, temperature and light conditions to those used in other successful mating experiments [63, 65]. Therefore, we think it is unlikely that our experimental conditions were the reason for this absence of mating and suggest that the two strains of *P*. *hallegraeffii* isolated during the current study are most likely reproductively isolated from other *Pseudo-nitzschia* species but are of the same mating type.

In Australian coastal waters only three species of *Pseudo-nitzschia* have been found to produce domoic acid thus far: *P*. *australis*, *P*. *cuspidata* and *P*. *multistriata* [26, 29]. Whilst historically it was assumed that the ‘*P*. *delicatissima* complex’ was the more non-toxic species group, this is no longer the case, with *P*. *cuspidata* being responsible for a significant toxic bloom in south eastern Australia in 2010, where maximum cell densities of > 6 x 10^6^ cells L^-1^ and DA in oyster tissue of 34 mg DA kg^-1^ were reported [26]. *P*. *hallegraeffii* sp. nov. isolates grown and harvested during their late stationary phase in our study did not produce detectable domoic acid concentrations however. Nevertheless, we do not discount that this species may prove to be toxic in future experiments or field scenarios, as DA production has been shown to vary with differing growth phases, cell sizes, physico-chemical parameters such as limiting nutrients, reproductive status, phycospheric bacterial communities and/or interactions with zooplankton [5, 66].

*P*. *hallegraeffii* sp. nov. was isolated from a relatively warm, low nutrient, diatom dominated phytoplankton community within the East Australian Current region. The first taxonomic examination of *Pseudo-nitzschia* collected from the Coral Sea and the EAC revealed five species belonging to the genus *Pseudo-nitzschia*: *P*. *fraudulenta*, *P*. *pseudodelicatissima*, *P*. *turgidula*, *P*. *lineola* and *P*. *subpacifica* [27]. Since this time *P*. *pseudodelicatissima* has been separated into eight species, the most likely result being that diversity has been previously underestimated in this region [26]. Furthermore, strains identified as *P*. *turgidula* by Hallegraeff [27] were most likely *P*. *dolorosa* [11]. Since this detailed examination there has been no further investigation into the diversity of *Pseudo-nitzschia* in tropical/subtropical Australian waters. We advocate that the discovery of a novel species by means of our eukaryotic microbial sampling suggests that even further diversity within the *Pseudo-nitzschia* genus, and other genera, is likely in eastern Australia. Moreover, the changing structure of the EAC, including its increasing strength and southward extension, suggests we can also expect further changes in species dispersal and connectivity along this coastline, ultimately influencing the ecology of phytoplankton in this region.

## Supporting information

S1 Fig Predicted ITS2 structures of A. *P*. *simulans* MC984 (GenBank accession number: MF374772) and B. *P*. *dolorosa* 300 (GenBank accession number: DQ336153).
(TIF)

S1 Table List of *Pseudo-nitzschia* clones used for phylogenetic reconstruction and for inferring *p*-distances (sequences obtained from Genbank, NCBI). (DOCX)

S2 Table Genetic distance (pairwise uncorrected *p*-distance) based on ITS-5.8S sequences. (XLS)

S3 Table Genetic distance (pairwise uncorrected *p*-distance) based on D1-D3 LSU rDNA sequences. (XLS)
