# Supplementary material for: A new diatom species P. hallegraeffii sp. nov. belonging to the toxic genus Pseudo-nitzschia (Bacillariophyceae) from the East Australian Current
Source: PLoS One. 2018 Apr 12;13(4):e0195622. doi: 10.1371/journal.pone.0195622 (PMC5896966; doi:10.1371/journal.pone.0195622)
Supplement: S1 Table — (DOCX) [file pone.0195622.s002.docx]

Supporting Table 1. List of *Pseudo-nitzschia* clones used for phylogenetic reconstruction and for inferring *p*-distances (sequences obtained from Genbank, NCBI).

| **Species** | **Strain ID** | **LSU rDNA** | **ITS rDNA** |
| --- | --- | --- | --- |
| *P. abrensis* | PnMi19 | KR021353 | – |
|  | Ner-J3 | KP172232 | – |
| *P. americana* | CV2 | U41390 | – |
|  | Kervel | – | EU523099 |
| *P. australis* | CV18 | U41393 | – |
|  | PLYSt19A | – | AY452527 |
| *P. batesiana* | PnTb19 | KC147534 | – |
| *P. bipertita* | PnMi04 | KR021334 | KR021318 |
| *P. brasiliana* | PnSm20 | HQ111371 | – |
|  | PnSm07 | – | HQ111404 |
| *P. caciantha* | AL-56 | DQ813812 | DQ813834 |
| *P. calliantha* | AL-112 | DQ813815 | DQ813841 |
| *P. circumpora* | PnSb58 | KC147533 | – |
| *P. cuspidata* | AL-17 | DQ813809 | – |
|  | Sydney1 | – | AY257862 |
| *P. decipiens* | Mex13 | EF506608 | DQ336156 |
|  | PnKk38 | KP337356 | KP337355 |
| *P. delicatissima* | AL-22 | DQ813810 | – |
|  | Tasm10 | – | AY257848 |
| *P. dolorosa* | PnKk08 | HQ111396 | – |
|  | 300 | – | DQ336153 |
|  | AL-59 | DQ813813 | – |
|  | BP3 | – | DQ336151 |
| *P. fukuyoi* | PnTb25 | KC147535 | KC147516 |
| *P. fraudulenta* | Limens1 | AF417647 | AY257840 |
| *P. fryxelliana* | NWFSC 241 | JN050296 | JN050288 |
| *P. galaxiae* | Mex23 | AY081136 | AY257850 |
|  | Sydnex4 | AY081137 | DQ336158 |
| ***P. hallegraeffii* sp. nov.** | **CTD44_2** | **MF044022** | **MF044023** |
|  | **CTD44_3** | **MF044024** | **MF044025** |
| *P. hasleana* | NWFSC 252 | JN050299 | JN085962 |
| *P. inflatula* | no7 | AF417639 | DQ329204 |
| *P. kodamae* | PnPd36 | KF482045 | KF482053 |
|  | PnMi92 | KR021339 | KR021310 |
| *P. limii* | PnMi16 | KR021343 | KR021311 |
| *P. lineola* | NWFSC 188 | JN050300 | JN091756 |
| *P. lundholmiae* | PnTb10 | KC147538 | KC147523 |
| *P. mannii* | AL-101 | DQ813814 | DQ813839 |
| *P. micropora* | PnKk14 | JN252433 | JN252422 |
| *P. multiseries* | NWFSC 011 | AF440772 | – |
|  | mu3 | – | AY257844 |
| *P. multistriata* | KoreaA | AF417654 | AY257843 |
|  | PnMi07 | KR021344 | KR021314 |
| *P. plurisecta* | Hobart5 | – | AY257851 |
|  | Ner-A1 | – | – |
| *P. pseudodelicatissima* | P-11 | AF417640 | AY257854 |
| *P. pungens* | PnSb48 | HQ111384 | – |
|  | PnSb44 | – | HQ111413 |
| *P. sabit* | PnPd57 | KM400615 | KM400610 |
| *P. seriata* | PLYSt52B | AY452526 | AY452524 |
| *P. simulans* | MC984 | MF374777 | MF374772 |
|  | MC3038 | MF374778 | MF374773 |
| *P. subfraudulenta* | PnMi80 | KR021349 | – |
|  | PnMi82 | – | KR021301 |
| *P. subpacifica* | Zhenbo7B | AF417644 | – |
|  | RdA8 | AF417642 | AY257860 |
| *P. turgiduloides* | 124C | EF531709 | – |
|  | 3-19 | – | AY257839 |
| *Nitzschia microcephala* | SOM | – | KC759159 |
| *Nitzschia navis-varingica* | VSP974-1 | AF417675 | – |
| *Phaeodactylum tricornutum* | ND52 | FJ214162 | – |
